# Hereditary Metabolic Bone Diseases: A Review of Pathogenesis, Diagnosis and Management

**DOI:** 10.3390/genes13101880

**Published:** 2022-10-17

**Authors:** Nipith Charoenngam, Aryan Nasr, Arash Shirvani, Michael F. Holick

**Affiliations:** 1 Section Endocrinology, Diabetes, Nutrition and Weight Management, Department of Medicine, Boston University School of Medicine, Boston, MA 02118, USA; 2 Department of Medicine, Mount Auburn Hospital, Harvard Medical School, Cambridge, MA 02138, USA; 3 Department of Medicine, Faculty of Medicine Siriraj Hospital, Mahidol University, Bangkok 10700, Thailand

**Keywords:** metabolic bone disease, sclerosing disorders, osteopetrosis, hypophosphatemic rickets, vitamin D-dependent rickets, hypophosphatasia, achondroplasia, osteogenesis imperfecta, hereditary connective tissue disorder

## Abstract

Hereditary metabolic bone diseases are characterized by genetic abnormalities in skeletal homeostasis and encompass one of the most diverse groups among rare diseases. In this review, we examine 25 selected hereditary metabolic bone diseases and recognized genetic variations of 78 genes that represent each of the three groups, including sclerosing bone disorders, disorders of defective bone mineralization and disorder of bone matrix and cartilage formation. We also review pathophysiology, manifestation and treatment for each disease. Advances in molecular genetics and basic sciences has led to accurate genetic diagnosis and novel effective therapeutic strategies for some diseases. For other diseases, the genetic basis and pathophysiology remain unclear. Further researches are therefore crucial to innovate ways to overcome diagnostic challenges and develop effective treatment options for these orphan diseases.

## 1. Introduction

Bone is a vital organ that provides structural support of the body and plays an essential role in regulating mineral metabolism. The major components of bone include bone matrix and bone cells. Bone matrix, which makes up around 90% of bone volumes, consists of inorganic bone matrix (mainly calcium hydroxyapatite) and organic bone matrix (mainly type I collagen and glycoproteins, growth factors and proteoglycans) [1,2]. The major types of bone cells include the osteoprogenitor cells, osteoblasts, osteocytes and osteoclasts. Metabolism of the bone is driven by the cooperative activity between the bone-forming osteoblasts and bone-resorbing osteoclasts [3]. This process is controlled by multiple pathways including the fibroblast growth factors (FGFs), bone morphogenetic proteins (BMPs), wingless-type (Wnt) genes, runt-related transcription factor 2 (RUNX2) and osteoblast-specific transcription factor (OSX), receptor activator of the nuclear factor kappa-Β (RANK) pathways [3]. These pathways are directly and indirectly regulated by several hormones, cytokines and growth factors including but not limited to the parathyroid hormone (PTH), vitamin D, osteoprotegerin, sex hormones, fibroblast growth factor-23 (FGF-23), sclerostin and dickkopf-1 among others [3,4].

Hereditary metabolic bone diseases comprise one of the most diverse groups among rare diseases. These disorders are characterized by genetic abnormalities in skeletal homeostasis with or without abnormal circulating calcium, phosphate, vitamin D metabolites and other markers. In this review, we examine a number of selected hereditary metabolic bone diseases representing each of the three groups: (1) sclerosing bone disorders, (2) disorders of defective bone mineralization and (3) disorder of bone matrix and cartilage formation. For each condition, we review up-to-date causative genetic variations, pathophysiology, clinical, biochemical and radiographic manifestation and treatment. It is crucial to provide early and accurate diagnosis and management for patients with metabolic bone disorders to maintain growth, development and quality of life as well as prevent fractures and other metabolic complications. With the advances in molecular genetics and basic sciences during the past decade, a number of novel treatments have been introduced and proven effective in some diseases, such as burosumab, an anti-fibroblast growth factor-23 (FGF23) monoclonal antibody for hypophosphatemic rickets and asfotase alfa, a bone-targeted enzyme replacement therapy for hypophosphatasia. However, the genetic basis, pathophysiology and therapeutic strategies of some other diseases remain to be further investigated. It is hoped that further scientific advancement can be utilized to innovate ways to develop effective therapeutics of these orphan diseases.

## 2. Sclerosing Disorders

### 2.1. Osteopetrosis

#### 2.1.1. Overview and Pathogenesis

Osteopetrosis, also known as “marble bone disease”, refers to a group of rare, heritable metabolic bone diseases characterized by reduced osteoclastic bone reabsorption, resulting in high bone mineral density and poor structural integrity. This overly dense bone structure usually belies brittleness that predisposes to fractures [5]. The autosomal dominant forms of osteopetrosis (ADO) are generally more benign with adult onset and minimal disability or symptoms. The autosomal recessive type (ARO), on the other hand, appears in infancy with serious health consequences leading to a high mortality rate [6]. The incidence of the autosomal recessive type is estimated at around 1 in 200,000 live births and the autosomal dominant type occurs 1 in 20,000 individuals [7].

ARO can be further subdivided into two forms: osteoclast-rich osteopetrosis and osteoclast-poor osteopetrosis. In osteoclast-rich ARO, osteoclasts are abundant but not functional usually due to a loss-of-function mutation in the *TCIRG1*, *CLCN7*, *OSTM1*, *SNX10*, or *PLEKHM1* genes. These five genes encode for proteins involved in endosomal and lysosomal vesicular transport which in turn affect bone resorption [8,9]. In contrast, osteoclast-poor ARO involves a deficiency of osteoclasts due to disruptions in osteoclastogenesis. The genetic basis of this subtype are mutations in the gene *TNFSF11*, encoding the receptor activator of nuclear factor kappa-Β ligand (RANKL), and the gene *TNFRSF11A* encoding its receptor, RANK. In ADO, types I and II are distinguished based on the sites of increased bone density and susceptibility to fragility fractures. The causative genetic mutations of these two types also differ. ADO type I is caused by mutations in the *LRP5* gene, whereas ADO type II involves mutations in the *CLCN7* gene [8]. Other types of osteopetrosis include ARO with renal tubular acidosis, caused by carbonic anhydrase II deficiency [10], and X-linked osteopetrosis, lymphedema, anhidrotic ectodermal dysplasia and immunodeficiency, caused by NF-kappaB essential modulator stop codon mutation [11].

Analysis of bone tissue in osteopetrosis may reveal a normal or elevated number of osteoclasts depending on the type. In rare cases, the number of osteoclasts may be low. Small “islands” of calcified cartilage within trabecular bone is a unique marker for osteopetrosis. Widespread immature interwoven bone within the matrix is also a common finding [12].

#### 2.1.2. Clinical, Biochemical and Radiographic Presentation

Clinical manifestations of osteopetrosis varies among the types, as shown in Table 1. Common findings include abnormally high bone density, bone fragility and deformities, dental abnormalities and variable degree of bone marrow failure [6,13].

In severe cases of osteopetrosis, decreased serum calcium levels, increased serum parathyroid hormone and increased 1,25-dihydroxyvitamin D [1,25(OH)_2_D] concentrations may be observed. In addition, elevated serum osteoclastic tartrate-resistant acid phosphatase (TRAP), brain isoenzyme creatine kinase, aspartate aminotransferase and lactate dehydrogenase may be present in some patients [5]. 

The major radiographic indication of osteopetrosis is an increase in bone mass with thickening of both trabecular and cortical bone. Other common radiographic findings include parallel bands of dense bone which can give the appearance of “bone-within-bone”. This observation is most common in the pelvis, long bones, vertebrae, and phalanges [5]. The metaphysis may widen and create an “Erlenmeyer flask” deformity. Due to excessive bone mineralization, Tc-99 m bone scans may reveal fractures or minimal bone marrow space and osteomyelitis [14]. Other common abnormalities are found in skull development ranging from a thickened basal aspect to the narrowing of the cranial nerve foramina to the absence or minimal development of the mastoid and paranasal sinuses [15].

#### 2.1.3. Treatment

The mainstay treatment for osteopetrosis are multidisciplinary surveillance and symptomatic management, which include adequate calcium and vitamin D intake for treatment and prevention of hypocalcemia, neurological evaluations for developmental delay and seizure, ophthalmologic surveillance for optic nerve compression and routine dental care [7,16]. Hematopoietic stem cell transplant is indicated only in patients with severe ARO with bone marrow failure [6,13]. Small interfering RNA therapy for treatment of ADO type II has been under investigation in the preclinical phase and is expected to be a novel therapeutic strategy [17]. 

### 2.2. Progressive Diaphyseal Dysplasia

#### 2.2.1. Overview and Pathogenesis

Progressive diaphyseal dysplasia, also known as Camurati-Engelmann disease, is a rare autosomal dominant hereditary disorder characterized by periosteal and endosteal thickening of long bone diaphysis. The genetic basis of this condition is a mutation in the gene that encodes for transforming growth factor-β1 (TGF-β1). The most prevalent mutation involves a substitution of arginine for cysteine at codon 218 (R218C) which affects the latency-associated domain of TGF-β1. This mutation may destabilize the peptide complex and hyperactivate the TGF signaling pathway, resulting in increased bone formation and resorption [18,19]. The skeletal thickening in progressive diaphyseal dysplasia occurs in a symmetric fashion and primarily affects the femur and tibia. Other long bones may be affected such as the radius, ulna, and fibula. Progressive diaphyseal dysplasia is categorized with abnormally hardened bone due to sclerosis causing the increase in bone mass [20].

Bone biopsies reveal bone formations along the diaphyseal surfaces. Woven bone matures and incorporates into cortical bone. In a case study of a young male with Camurati-Engelmann disease, histological examinations revealed a thickened periosteum in the femurs and tibias. Small vascular walls showed substantial thickening and dense compact structures were seen in the bone cortex [21].

#### 2.2.2. Clinical, Biochemical and Radiographic Presentation

Clinical manifestations of progressive diaphyseal dysplasia include patients intense bone pain, myopathy causing muscle weakness, stiff joints causing pain and immobility when walking and fatigability with onset of symptoms ranging from early childhood to late adulthood [20]. These patients often have a characteristic waddling gait. Loss of vision and/or hearing can occur if the bones in the skull are significantly affected. The limbs may become slim due to the loss of muscle mass, but the bones remain prominent and tender. The skull may also be affected which manifests as an enlarged cranium, prominent forehead, proptosis, cranial nerve palsy, and hydrocephalus [20]. Common laboratory findings associated with this condition are hypocalcemia and hypocalciuria associated with increased renal calcium reabsorption, which represent marked positive calcium balance. Elevated bone turnover markers (i.e., serum alkaline phosphatase and urine hydroxyproline excretion) can occur. In addition, some patients can experience connective tissue inflammation which manifests as mild anemia and elevated erythrocyte sedimentation rate and leukopenia [22]. 

Affected areas of progressive diaphyseal dysplasia display increased radiotracer uptake under Tc-99 m bone scans. Hyperostosis of both endosteal and periosteal surfaces are usually irregular and patchy. They spread symmetrically along the diaphysis and can even extend to the metaphysis of long bones [22]. 

#### 2.2.3. Treatment

Corticosteroids can be administered to relieve bone pain and help improve muscle weakness and fatigue [20,23]. In addition, treatment with losartan, an angiotensin II type 1 receptor antagonist, is also effective in pain relief and improvement of muscle function in some individuals. This is due to the downregulation of TGF-β1 signaling by losartan [20,24,25]. There has been efforts to develop drugs to directly inhibit TGF-β signaling; however, clinical trial data are still unavailable [26,27]. Clinical data on outcomes of TGF-β inhibitor could enlighthen new treatment approaches for progressive diaphyseal dysplasia.

### 2.3. Melorheostosis

#### 2.3.1. Overview and Pathogenesis

Melorheostosis is an extremely rare sclerosing bone dysplasia caused by mesenchymal dysplasia, the disorder is usually unilateral and monozstotic (affecting a single bone) and the X-ray appearance of the affected bone resembles dripping wax from a melting candle. The disease typically affects the appendicular skeleton in a limited segmental fashion. Melorheostosis may affect both cortical bone and the adjacent soft tissue [28,29]. LEM domain-containing protein 3 (*LEMD3*) gene mutations have been correlated with several familial cases of melorheostosis. However, the correlation is much stronger with other hereditary dysplasias, such as osteopoikilosis. Therefore, modern literature does not consider *LEMD3* mutations to be the cause of melorheostosis [29,30]. In fact, the majority of patients with melorheostosis display no LEMD3 abnormalities. Recent studies have shown that most cases arise from somatic *MAP2K1* mutations, while a minority of cases can arise from mutations in related pathways, such as KRAS. The classic “dripping candle wax” appearance of bone is more often correlated with the somatic *MAP2K1* mutation [31,32]. Melorheostosis causes endosteal thickening during childhood and periosteal bone formations in adulthood. Sclerosis appears with thick and irregular lamella. A case study of 15 patients with melorheostosis revealed a prominence of dense cortical bone, woven bone, hypervascular features and increased porosity. Prominent cement lines were seen in 5 of those patients [33]. 

#### 2.3.2. Clinical, Biochemical and Radiographic Presentation

Common clinical presentations of melorheostosis include pain, bony swelling, joint and limb deformities, localized growth abnormalities, limited motion, numbness, and weakness (Table 1). It has also been associated with a benign inner ear osteosclerosis. The consensus is that the appendicular skeleton is most commonly affected, however, this could also be due to the elusive diagnosis of such features within the axial skeleton [28]. There is currently no definitive laboratory findings used for the diagnosis of melorheostosis [34]. Serum calcium, phosphorus, and alkaline phosphatase appear normal in most cases.

On radiographs, dense hyperostosis of both the periosteal and endosteal surfaces of bones can be observed. These bones are usually located in the lower extremities but can affect other areas as well. The soft tissue surrounding the lesions or joints may display ectopic bone. In addition, high blood flow in the lesions can be spotted through the accumulation of tracers on bone scans [35,36].

#### 2.3.3. Treatment

Non-surgical interventions include anti-inflammatory medications, bisphosphonates, nifedipine, physical therapy, braces, castings, nerve block, and sympathectomies. These treatments are generally ineffective in treating limb deformities. Surgical interventions are usually unsuccessful due to high rates of recurrent deformities [34]. 

### 2.4. Pyknodysostosis

#### 2.4.1. Overview and Pathogenesis

Pyknodysostosis from the Greek word punknos meaning dense is a rare autosomal recessive geneic disorder causing dense bone that is structurally unsound causing fractures. This disorder is caused by an exon 5-homozygous mutation in the *CTSK* gene that encodes for cathepsin K, a lysosomal metalloproteinase which plays an important role in bone matrix degradation by osteoclasts [37]. This disease is characterized by a decreased activity of osteoblasts and osteoclasts resulting in short-limbed short stature, dysmorphic facial features, osteosclerosis, bone fragility and fractures, and dental and nail abnormalities [37,38]. In addition, histological analysis reveals the osteoclasts containing large vacuoles with undigested collagen that form demineralized matrix borders on the surface of the bone [39].

#### 2.4.2. Clinical, Biochemical and Radiographic Presentation

Common clinical presentations of pyknodysostosis are short stature, dysmorphic facial features, kyphoscoliosis, chest deformity, high arched palate, proptosis, and blue sclera (Table 1). Other clinical features include wrinkled skin, nail abnormalities, and sleep apnea [40]. Laboratory testing in pyknodysostosis reveals a normal serum calcium, phosphate, alkaline phosphatase, and vitamin D. Growth hormone deficiency and low insulin-like growth factor-1 level are also common findings [41,42].

Radiographs of patients with pyknodysostosis usually reveals increased bone density, generalized progressive osteosclerosis of the long bones, acroosteolysis of the distal phalanges, non-pneumatized mastoids, delayed fusion of cranial sutures, obtuse mandibular angle, clavicular dysplasia, and susceptibility to fractures. Long bones are generally normally shaped but with narrowed medullary cavities. Sclerosis of the calvarium and the base of skull may also be present. A common dental abnormality is the persistence of primary teeth [38].

#### 2.4.3. Treatment

There is no known definitive treatment for pyknodysostosis but symptoms can be managed across a multidisciplinary treatment approach [38]. Surgical intervention may be required to treat fractures, craniofacial and spine abnormalities. Growth hormone therapy can treat GH deficiency and short stature while restoring body proportions [41,42]. Obstructive sleep apnea can be managed with surgical treatment or ventilatory support [43]. Dental anomalies should be treated under the supervision of orthodontists [38,44].

### 2.5. High Bone Mass Associated with LRP5 and LRP6 Mutations

#### 2.5.1. Overview and Pathogenesis

Low-density lipoprotein receptor-related protein 5 (LRP5) is a regulator of bone mineral density by acting as the Wnt/β-catenin signaling pathway receptor [45]. Specific autosomal dominant mutations of the *LRP5* gene has been shown reduce binding affinity of the protein with dickkopf-1 and sclerostin, inhibitors of bone formation [46]. This subsequently result in high bone mineral density and sclerosing skeletal abnormality. Low-density lipoprotein receptor-related protein 6 (LRP6) plays an essential role in activation of Wnt/β-catenin signalling by acting as a co-receptor with LRP5. Missense mutations of *LRP6* is expected to have similar consequences on bone metabolism to LRP5 mutations.

#### 2.5.2. Clinical Presentation

High bone mass associated with *LRP5* mutation has a variable phenotype and may be asymptomatic. Patients present with extremely high bone mineral density and can have increased calvarial thickness, craniosynostosis, striking square jaw, torus palatinus and thickened cortices of long bone [46,47,48]. 

In 2019, heterozygous *LRP6* mutations were identified in two american families with high bone mass phenotype and absence of adult maxillary incosors [49]. Other overlapping clinical features with patients with *LRP5* mutations include broad jaw, torus palatinus and teeth encased in bone. In addition, there has been a report of diffuse thickening of the skull, optic nerve dilatation and narrowing of optic and autidory canals [50]. 

#### 2.5.3. Treatment

There is no specific treatment for these conditions. Management is mainly symptomatic.

### 2.6. Pyle Disease 

#### 2.6.1. Overview and Pathogenesis

Pyle disease (metaphyseal dysplasia) is an extremely rare autosomal recessive skeletal disorder causing widening metaphysis first described by Edwin Pyle in 1931 on a on a 5-year-old boy who presented with severe genu valgum in both knees and cranial deformities [51]. This condition is caused by loss-of-function mutations of the *SFRP4* gene, which involves in skeletal remodeling by suppressing the canonical and non-canonical Wnt signaling pathway [52,53]. 

#### 2.6.2. Clinical and Radiographic Presentation

The major clinical feature of Pyle disease is genu valgum and the key radiological finding is massive metaphyseal expansion extending into meta-diaphyseal junction, also known as “Erlenmeyer flask” deformity [54]. Broadening of proximal two-third humeral bones and distal two-thirds radial and ulnar bones can also be observed. Other clinical presentations include metaphyseal fracture, bilateral knee enlargement, broadening proximal phalanges and distal metacarpal bones, dental abnormalities and jaw protrusion (prognathism) [55]. 

#### 2.6.3. Treatment

Given the benign nature of the disease, most cases of Pyle disease do not require intervention. However, orthopedic treatment may be required for significant genu valgum or metaphyseal fractures [55].

### 2.7. Hyperostosis Corticalis Generalisata

#### 2.7.1. Overview and Pathogenesis

Hyperostosis corticalis generalisata, also known as van Buchem’s disease, was first described by van Buchem et al. in 1955. This autosomal recessive disorder is caused by a duplication or deletion of regions within the *SOST* gene that encodes sclerostin, a negative regulator of bone formation synthesized by osteocytes [56]. This condition is characterized by endosteal hyperostosis of the mandible, skull, ribs, clavicles, and diaphysis of long bones [57]. Van Buchem’s disease has therefore been classified as a craniotubular hyperostosis [58]. Most patients come from a small fishing village in the Netherlands [59]. Histological examinations in hyperostosis corticolis generalisata demonstrate increased osteoblastic bone formation and reduced osteoclastic resorption leading to thickened trabeculae and osteoid accumulation [58]. 

#### 2.7.2. Clinical and Biochemical Presentation

The most prominent features of van Buchem’s disease include an enlarged mandible and thickened skull which can lead to facial nerve palsy, hearing loss, and optic atrophy [58]. Blood tests reveal an elevated bone-specific alkaline phosphatase, indicating increased bone formation. Serum calcium and phosphorus are generally normal but sclerostin levels may be low [60]. Radiographic findings of this condition include endosteal thickening that usually narrows the medullary cavity and creates dense diaphyseal cortical bone. Increased thickening of long bones, ribs, skull, and mandible are often observed [59].

#### 2.7.3. Treatment

Surgical intervention may reduce the neurological symptoms caused by cranial nerve compression. However, the progression of hyperostosis is currently untreatable. 

### 2.8. Sclerosteosis

#### 2.8.1. Overview and Pathogenesis

Sclerosteosis is a rare sclerosing bone disorder characterized by progressive generalized osteosclerosis. This condition has been linked to autosomal recessive loss-of-function mutations in the *SOST* gene, encoding the protein sclerostin, which inhibits osteoblastic activity and suppresses bone formation by antagonizing the Wnt/β-catenin signaling pathway [61,62]. It can also be caused by heterozygous or homozygous missense mutations in the *LRP4* gene, which encodes a Wnt signaling coreceptor that facilitates the action of sclerostin on the Wnt/β-catenin pathway [63,64]. 

#### 2.8.2. Clinical Presentation

Patients with sclerosteosis present with bone overgrowth throughout the life affecting primarily the skull, mandible and the tubular bones. Enlargement of the jaw or facial bones can result in facial abnormality, cranial nerve impingement and increased intracranial pressure [65,66]. Syndactyly and tall stature can also be observed and are major distinguishable features from hyperostosis corticalis generalisata [66].

#### 2.8.3. Treatment

There is no specific treatment for sclerosteosis. The management approach is decompression of cranial nerves, surgical correction of syndactyly and reduction of mandibular overgrowth [27].

### 2.9. Juvenile Paget’s Disease

#### 2.9.1. Overview and Pathogenesis

Juvenile Paget’s disease, also known as osteitis deformans, is a rare genetic disorder characterized by rapid bone turnover which occurs in early childhood. The main cause of Juvenile Paget’s disease is a mutation in the *TNFRSF11B* gene, which encodes for osteoprotegerin (OPG). Analyzing familial patterns demonstrates an autosomal dominant pattern of inheritance with variable penetrance [67,68,69]. Patients with juvenile Paget’s disease have immature osseous tissue with weak and disorganized woven bone. Osteoblast and osteoclast numbers are significantly increased, and the bone cortices are typically widened. Histomorphometric images of the iliac crest trabecular bone also reveals distinct patterns such as abnormal series of parallel trabecular plates [67].

#### 2.9.2. Clinical, Biochemical and Radiographic Presentation

The clinical manifestations of Juvenile Paget’s disease can be both skeletal and extra-skeletal. Skeletal presentations are progressive bone deformities, while extra-skeletal manifestations can include hearing loss, retinopathy, vascular calcification and internal carotid artery aneurysm formation (Table 1) [67,70]. Biochemical analysis reveals a significantly high level of alkaline phosphatase (usually 5–20 fold increase). Serum calcium, phosphate, and RANKL are generally elevated. PTH, periostin, and sclerostin may be elevated or normal [71,72].

Findings: Radiography initially reveals expanded long bones with osteopenic cortices. Eventually, the long bones become bowed with diffuse osteosclerosis and hyperostosis. The skull appears enlarged with marked prominence of the frontal and parietal areas. Kyphosis and scoliosis may or may not be present in affected individuals [67,73].

#### 2.9.3. Treatment

There is currently no known definitive treatment for Juvenile Paget’s disease. However, case reports have demonstrated successful outcomes with the administration of antiresoptive medications, including calcitonin, bisphosphonates, recombinant osteoprotegerin, and denosumab [67,73,74,75,76].

### 2.10. Paget’s Disease

#### 2.10.1. Overview and Pathogenesis

Paget’s disease is characterized by abnormally high bone turnover which leads to a disorganized bone remodeling [77]. It affects 1–2% of the population and is the second most common bone disease after osteoporosis. Most of these patients are over the age of 55 [78]. There has been speculation that Paget’s disease is caused [79] by a slow virus. However, the inheritance pattern of Paget’s disease is autosomal dominant in 15–40% of the cases. In the remaining cases, the inheritance pattern of Paget’s disease is unclear [80]. Mutations in the *SQSTM1* gene is the most common genetic cause of this disease. Analysis of different populations reveal that the most common type of mutation is pP392L. However, other types of mutations have also been identified. These mutations usually affect the ubiquitin-associated domains of the proteins [81]. Other genes associated with risk of Paget’s disease include *TNFRSF11A* (encoding RANK) and *TNFRSF11B* (encoding OPG) [82,83,84]. Bone biopsy in Paget’s disease reveals a disorganized and poorly structured bone matrix. Instead of a healthy lamellar bone mineralization, the collagen fibers are randomly oriented and have a woven appearance. Increased vasculature and numerous abnormal osteoclasts are also visible [85].

#### 2.10.2. Clinical, Biochemical and Radiographic Presentation

Paget’s disease, which may involve a single bone but often involves more than one bone, is sometimes accompanied by bone pain, bone fractures and bone deformity especially when a lower limb is involved. Due to the rapid localized bone turnover there is increased blood circulation to the affected bone(s). Usually when more than 40% of the skeleton is involved this can cause a significant, up to 10%, increase in cardiac output. This can lead to high output cardiac failure in patients with a compromised cardiovascular status. The localized increased blood flow can cause increased warmth over the affected bone. On physical exam this can be detected by feeling increased warmth in the affected area that can be detected on physical exam by touching the area with an open palm or back of the hand (which is the most sensitive area to detect changes in temperature). This can be a valuable diagnostic tool not only for detecting areas of pagetic bone but also during treatment if the affected area feels the same temperature as a surrounding area it is likely that the treatment is successful. This is especially helpful in rural areas where X-rays and bone scans are not easily available. Most patients are asymptomatic and often are diagnosed based on the finding of an island woven bone in the pelvis as an incidental finding. However, if the Pagetic bone is close to a joint such as the hip or knee this can cause osteoarthritis. If the bone involvement is near a foramen this can cause nerve compression especially in the spine and neuropathy. Involvement of the skull can lead to headache and if near the 8th nerve foramen can cause not only hearing loss and tinnitus but also vertigo. In rare cases especially in the spine the Pagetic bone can develop into an osteosarcoma. Elevated serum alkaline phosphatase, indicating increased bone formation, is a common laboratory finding of Paget’s disease. An elevation in bone specific alkaline phosphatase helps to rule out other causes for an elevated alkaline phosphatase. In bone remodeling markers including serum osteocalcin, a bone formation marker, serum C-telopeptide CTX) and urine N-telopeptide (NTx) are also elevated [77].

On Tc-99 bone scan, there is increased uptake in regions of increased osteoblastic activity and vasculature [81]. However, increased osteoblastic activity may also be associated with other bone diseases. Therefore, this observation is not just specific to Paget’s disease. CT scans can provide valuable details used in the diagnosis of this disease. Trabecular and cortical thickening, osseus expansion and osteolysis are common observations. In addition, vertebral sclerosis may also be detected [86].

#### 2.10.3. Treatment

Anti-resorptive medications can be used to treat Paget’s disease including calcitonin and bisphosphonates. Bisphosphonates are first-line therapy for this disease. Zoledronic acid is often used for active disease and can be effective for up to 2–3 years [87]. Denosumab is also reported to be effective in normalizing biochemical marker but not bone scintigraphy in few cases [88]. Surgical intervention may be necessary in treating bone deformities associated with Paget’s disease. Symptomatic treatment with analgesics can help alleviate bone pain [77,78].

### 2.11. Osteopathia Striata

#### 2.11.1. Overview and Pathogenesis

Osteopathia striata with cranial sclerosis (OS-CS) is a X-linked dominant genetic disorder characterized by linear striations within the metaphyseal areas of long bones. This condition is caused by mutations in the *WTX* gene, a repressor of the WNT signaling pathway [89]. This can be caused by germline deletions of WTX gene or truncating point mutations within the gene [90]. WTX protein is also a tumor suppressor and its inactivation have been associated with cancer. However, there have been no indication of increased cancer risk among patients with OS-CS. Cranial histology in OS-CS indicates hyperactive periosteal bone formation which may potentially cause cranial sclerosis. However, the histological basis behind this sclerosis of the skull and the linear striations of the long bones still remains a mystery [91].

#### 2.11.2. Clinical and Radiographic Presentation

Clinical signs of OS-CS are macrocephaly, characteristic facial features (frontal bossing, hypertelorism, depressed nasal bridge, prominent mandible, and epicanthal folds), hearing loss, orofacial clefting, and mild developmental delay (Table 1). Mildly affected males are more prone to congenital and musculoskeletal defects. However, severe cases in males can lead to multiple-malformation syndrome and significant pre- and postnatal lethality [92].

Radiographic studies of OS-CS reveal sclerosis of the cranium, lamellar and trabecular bones. Other common findings include metaphyseal, longitudinal striations of the pelvis and long bones which may be absent in mildly affected males. Fibular aplasia or hypoplasia and small exostoses may also be found [92].

#### 2.11.3. Treatment

There is no known definitive treatment for OS-CS. However, a multidisciplinary approach to symptom management is a viable option, including surgery for cranial nerve compression and physical therapy for musculoskeletal complications [92,93]. 

### 2.12. Osteopoikilosis

#### 2.12.1. Overview and Pathogenesis

Osteopoikilosis is a rare autosomal dominant bone dysplasia caused by a loss-of-function mutation in the LEM domain containing 3 (*LEMD3*) gene which encodes for an inner nuclear membrane protein [94,95]. This condition generally benign and free from any major symptoms. It is characterized by numerous bone islands that appear on X-ray as numerous white densities that usually affect the appendicular skeleton. These formations are caused by a failure of resorption of secondary spongious bones [95]. Histological examination reveals bone “islands”. These foci are numerous and represent dense trabeculae of spongy bone within the spongiosa [96].

#### 2.12.2. Clinical and Radiographic Presentation

Diagnosis of osteopoikilosis is usually incidental through radiography taken for other medical reasons and can mimic other skeletal diseases, including mastocytosis, melorheostosis, tuberous sclerosis, and osteoblastic metastasis [97,98]. It is generally benign and asymptomatic; however, a minority of patients may experience mild articular pain and joint effusions [94].

Osteopoikilosis has characteristic bony sclerosis on X-ray that appear in the epiphysis and metaphysis of long bones, skull, vertebrae, or ribs. They are usually hyperdense, small, abundant, and round or oval-shaped. These lesions are normally symmetric and range in size from a few millimeters to several centimeters. Other commonly affected sites are the phalanges, carpals, and metacarpals of the hands. X-rays of both hands can usually provide a definitive diagnosis of osteopoikilosis. Bone scanning radiotracers are not taken up by osteopoikilosis lesions [36,95,99].

#### 2.12.3. Treatment

Most cases of osteopoikilosis are asymptomatic and therefore require no treatment. However, in cases where pain is present, NSAIDs and analgesics, such as acetaminophen and opioids, may be used [95]. 

## 3. Disorders of Defective Bone Mineralization

### 3.1. Hypophosphatasia

#### 3.1.1. Overview and Pathogenesis

Hypophosphatasia (HPP), Rathbun’s syndrome, is a genetic disorder caused by genetic mutations of the *ALPL* gene that encodes tissue-nonspecific-isoenzyme of alkaline phosphatase (TNSALP) [100,101,102,103,104]. TNSALP is a phosphohydrolase enzyme expressed in multiple organs including the skeleton, developing teeth, liver and kidney [102,103,104]. In the bone, TNSALP converts inorganic pyrophosphate into inorganic phosphate, which is essential for crystallization of calcium hydroxyapatite. Impaired calcium hydroxyapatite formation in HPP leads to defective bone mineralization [102,103,104]. Accumulation of circulating inorganic pyrophosphate can increase the risk for developing calcium pyrophosphate deposition (CPPD) in adults [105]. Additionally, pyridoxal 5′-phosphate is another substrate of TNSALP. Thus, severe HPP can cause impaired γ-carboxyglutamic acid synthesis in the central nervous system and therefore precipitates pyridoxine-dependent seizures [106]. 

#### 3.1.2. Clinical and Biochemical Presentation

To date, over 400 different variants in the 12 exons of the *ALPL* gene have been have identified to be causative variants for HPP [107]. This variety of pathogenic variants explains the highly variable clinical expression of HPP, which can manifest in several forms with varying age onset and severity depending on the degree of enzyme deficiency. These forms include odonto HPP, adult HPP, childhood HPP, infantile HPP and perinatal HPP (ranked by increasing severity) [104]. The diagnostic hallmark of HPP is low serum ALP level with exclusion of secondary causes of low ALP (i.e., hypothyroidism, hypercortisolism, vitamin C deficiency and zinc and magnesium deficiency) [108]. Elevated urine pyridoxal 5′-phosphate is specific for HPP and can be observed in asymptomatic parents of affected children [109]. Serum calcium and/or phosphate can be elevated in some cases due to impaired sequestration into the skeleton.

Patients with the mildest form odoto HPP present with isolated premature loss of primary teeth without other skeletal abnormalities. Adult with HPP presents with osteomalacia and systemic CPPD deposition. Childhood HPP presents with premature loss of primary teeth, fragility fractures and rachitic skeletal deformities, which may improve in early adulthood after epiphyseal plates close. Infantile HPP manifests as severe rachitic deformities, chest wall deformity, fragility fractures, tracheomalacia, brachycephaly, hypertelorism, skull deformity causing increased intracranial pressure, pyridoxine-dependent seizure and hypercalcemia due to inability to deposit calcium in the skeleton. Infantile HPP, the most severe form, can cause in utero limb deformity, cardiopulmonary failure, intracranial hemorrhage and myelophthisic anemia [101,110,111,112,113]. Notably, diagnosis of pediatric forms of HPP is believed to be often missed as this condition shares signs and symptoms with other metabolic bone disorders and most laboratories report only reference of serum ALP for adults or without lower limit. 

#### 3.1.3. Treatment

The specific therapy of HPP is asfotase alfa, a bone-targeted enzyme replacement therapy that is shown to improve survival and clinical outcomes of HPP [114,115]. In mild forms of HPP, asfotase α is not indicated. Adequate vitamin D and calcium intake should be maintained to improve bone mineralization. However, oversupplementation should be avoided and serum calcium and phosphate and urinary calcium should be monitored closely to avoid nephrocalcinosis and systemic calcification [112,116]. Vitamin B6 administration may be of benefit for prevention of pyridoxine-dependent seizure in infantile HPP [112,116,117]. 

### 3.2. Hypophosphatemic Rickets

#### 3.2.1. Overview and Pathogenesis

FGF23 is a peptide hormone secreted by the osteocytes. It inhibits the expression of sodium-phosphate cotransporters (NPT2a and NPT2c) in the proximal tubule, resulting in decreased proximal tubular phosphate reabsorption and therefore increased phosphaturia [118]. FGF23 also inhibits the expression of the 25-hydroxyvitamin D-1α-hydroxylase in the kidneys and enhances the expression of the 25-hydroxyvitamin D-24-hydroxylase, which, in turn, leads to decreased circulating 1,25(OH)_2_D concentrations, thereby causing decreased intestinal calcium and phosphate absorption [119]. The overall effect of FGF23 is to cause phosphate depletion state. 

Hypophosphatemic rickets is a spectrum of genetic disorder characterized by defective bone mineralization secondary to hypophosphatemia associated with excessive circulating FGF23. X-linked hypophosphatemic rickets (XLH) is the most common form of hypophosphatemic rickets that affects approximately 1:20,000 live births [120,121,122]. It is caused by loss-of-function mutation of the *PHEX* gene in the X chromosome that encodes a transmembrane endopeptidase responsible for catabolism of FGF23 [123]. The less common forms are autosomal dominant hypophosphatemic rickets (ADHR) and autosomal recessive hypophosphatemic rickets type 1 and 2 (ARHR1 and ARHR2). ADHR is caused by a mutation of the *FGF23* gene at the enzymatic cleavage site [124], thereby preventing its catabolism, whereas ARHR1 and ARHR2 are associated with *DMP1* and *ENPP1* mutations, respectively, both of which indirectly cause circulating FGF23 elevation [125,126].

#### 3.2.2. Clinical and Biochemical Presentation

Patients with hypophosphatemic rickets usually present after the age of 6 months with signs and symptoms similar to those of nutritional rickets. These include muscle weakness, waddling gait, growth retardation, delayed tooth eruption, dental abscess, frontal bossing, craniosynostosis, long bone deformities and thickened costochondral junctions [127,128]. The biochemical hallmarks of hypophosphatemic rickets are hypophosphatemic with increased urinary phosphate excretion, increased serum FGF23 and alkaline phosphatase and inappropriately low serum 1,25(OH)_2_D. Serum calcium and PTH are usually unaffected. Urinary calcium excretion can be low as a result of decreased serum 1,25(OH)_2_D which causes decreased intestinal calcium and phosphate absorption [129]. 

Hypophosphatemic rickets can be diagnosed based on clinical presentation with positive family history and biochemical characteristics, which can be confirmed by genetic testing panel for causative mutations. 

#### 3.2.3. Treatment

The mainstay treatment for hypophosphatemic rickets oral supplementation of phosphate and 1,25(OH)_2_D_3_ or 1α(OH)D_3_ along with adequate intake of calcium and vitamin D. Oversupplementation of calcium and active vitamin D medications should also be avoided as it can precipitate hypercalcemia and hypercalciuria causing kidney stone and nephrocalcinosis [127,130,131]. Potassium phosphate preparations are preferred over sodium phosphate as the latter provides high sodium load which can precipitate hypercalciuria. On the other hand, inadequate calcium, vitamin D and active vitamin D medications can cause secondary hyperparathyroidism which worsens phosphaturia. Recently, a novel human monoclonal anti-FGF23 antibody, burosumab, was approved for treatment of XLH in patients aged over 6 months [132,133,134,135,136]. Nonetheless, no specific recommendations exist to date for use of burosumab for other forms of hypophosphatemic rickets. 

### 3.3. Vitamin D-Dependent Rickets

#### 3.3.1. Overview and Pathogenesis

Vitamin D is a steroid molecule that maintains calcium and phosphorus homeostasis. Once entering the circulation via cutaneous synthesis or intestinal absorption, vitamin D gets metabolized by the hepatic enzyme 25-hydroxylase to 25-hydroxyvitamin D, which is then converted by the renal enzyme 25-hydroxyvitamin D-1α-hydroxylase into the active form 1,25-hydroxyvitamin D [1,25(OH)_2_D]. Activation of the intracellular vitamin D receptor by 1,25(OH)_2_D promotes intestinal calcium and phosphate absorption, renal tubular calcium reabsorption, and mobilization of calcium from the bone [137,138,139,140].

The common mechanism by which vitamin D deficiency and all types of vitamin D-dependent rickets causes bone mineralization defect is decreased serum ionized calcium secondary to impaired intestinal calcium absorption. This leads to a compensatory increase in the expression and secretion of parathyroid hormone, which subsequently causes phosphaturia by internalization of sodium-dependent phosphate cotransporter (NPT2a) in the renal proximal tubule and therefore decreased calcium-phosphate product to crystallize, i.e., mineralize the bone collagen matrix [13,138,139,140]. 

**Figure 1:**
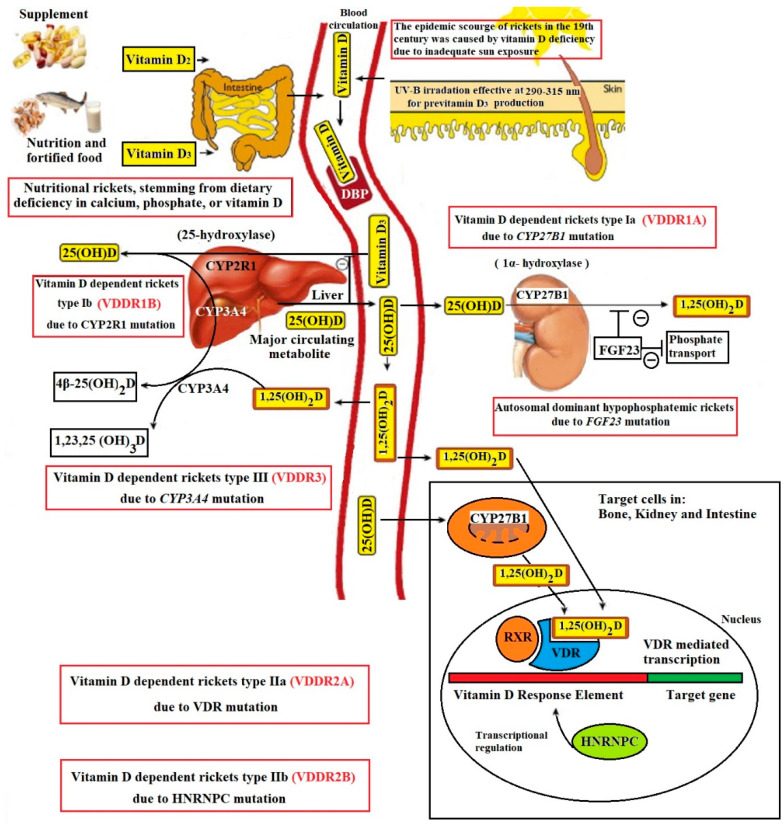
Schematic representation vitamin D metabolism and hereditary disorders affecting vitamin D pathway. Copyright Holick 2022.

Vitamin D dependent rickets type I, also known as pseudovitamin D deficiency rickets (PDDR), is caused by inactivating mutation of CYP27B1 encoding 1α-hydroxylase, which results in a defect in 1,25(OH)_2_D production. Vitamin D dependent rickets type II or hereditary vitamin D resistant rickets (HVDRR) is characterized by impaired recognition of 1,25(OH)_2_D due to a mutation of the vitamin D receptor (VDR) [140,141]. There has been one case report of rickets caused by abnormal expression of hormone responsive element-binding protein that binds to the vitamin D responsive element, thereby causing impaired genomic response to activation of VDR by 1,25(OH)_2_D [142]. The recently identified vitamin D dependent rickets type III is noted to be due to a mutation of the *CYP3A4* gene that causes a marked increase in the catabolism of 1,25(OH)_2_D [143] (Figure 1). 

#### 3.3.2. Clinical and Biochemical Presentation

Patients with PDDR present in their first year of life with severe hypocalcemia causing hypocalcemic tetany, seizure, laryngospasm and cardiomyopathy as well ashypophosphatemia, secondary hyperparathyroidism, high serum alkaline phosphatase and undetectable or inappropriately low serum concentrations of 1,25(OH)_2_D. Unlike those with vitamin D deficiency rickets, their serum 25(OH)D are usually normal [140]. These patients have similar skeletal manifestations as seen in those with nutritional vitamin D deficiency rickets, which are thought to be due to defects in both bone mineralization and chondrocyte maturation. These include proximal muscle weakness, waddling gait, short stature, delayed tooth eruption, sweating, craniotabes, frontal bossing, widened fontanelles, rachitic rosary, sternal protrusion, flattened pelvic bones, bowing deformities of arms and legs and flattened pelvic bones [140]. 

Patients with HVDRR have all the clinical and biochemical presentations of PDDR except for that they usually have low serum 25(OH)D levels and markedly high serum 1,25(OH)_2_D. In addition, certain VDR mutations in HVDRR can lead to progressive alopecia beginning in the first year of life. The reported patient with abnormal expression of hormone responsive element-binding protein had similar presentation of HVDRR including alopecia [142]. Patients with vitamin D dependent rickets type III present with clinical and radiological presentation of rickets and low serum 25(OH)D and 1,25(OH)_2_D concentrations [143]. 

#### 3.3.3. Treatment

Patients with PDDR respond well to adequate calcium intake and physiologic dose of 1,25(OH)_2_D_3_ (1–2 µg/day). Once treatment is initiated, serum calcium is expected to improve in 24 h and radiologic evidence of healing rickets can be observed within 3 months. On the other hand, patients with HVDDR have variable responses to physiologic dose of 1,25(OH)_2_D_3_ therapy depending on the degree of resistance. Some patients may need higher doses of 1,25(OH)_2_D_3_. Others may have complete resistance to the therapy and require intravenous calcium and phosphate infusion [140,141,144]. 

### 3.4. Axial Osteomalacia

#### 3.4.1. Overview and Pathogenesis

Axial osteomalacia is characterized by selective demineralization of axial skeleton. This rare condition is thought to be caused by loss of function of osteoblasts causing coarsened trabeculae and increased cortical and total bone volume with excessive osteoid [145]. Most of the reported cases are sporadic but mother-to-son transmission of axial osteomalacia has been reported, suggesting a hereditary disorder [145]. However, no genetic mutation has been identified in any of the family cluster. 

#### 3.4.2. Clinical and Biochemical Presentation

Axial osteomalacia often affects middle-aged or older men presenting with bone pain along the axial skeleton, most commonly the cervical spine. Pathologic coarsening of trabecular bone of the axial skeleton (i.e., pelvis and spine) consistent with features of osteomalacia can be observed on plain radiographs [145]. Additionally, findings of ankylosing spondylitis have been reported [146]. The biochemical manifestation of this condition is nonspecific with normal or mildly elevated serum alkaline phosphatase and otherwise normal serum concentration of calcium, phosphate, PTH, 25(OH)D and 1,25(OH)_2_D. 

#### 3.4.3. Treatment

Given its unknown etiology and benign course, the management approach of axial osteomalacia is symptomatic treatment with pain control. Vitamin D and calcium therapy has been shown to reverse the disease process [145].

## 4. Disorders of Bone Matrix and Cartilage Formation

### 4.1. Achondroplasia

#### 4.1.1. Overview and Pathogenesis

Achondroplasia, commonly associated with dwarfism, is the most common hereditary cause of disproportionate short stature affecting approximately 1/15,000 to 1/40,000 of the population [147]. This condition is an autosomal dominant genetic disorder arising from a gain-of-function mutation of the fibroblast growth factor receptor 3 (FGFR3) gene that leads to impaired endochondral ossification due to defective chondrocyte proliferation [148,149]. 

#### 4.1.2. Clinical Presentation

Genetic disorders associated with gain-of function FGFR3 can be categorized into 4 categories with different severity: achondroplasia, hypochondroplasia and type I and II thanatophoric dysplasia [149,150]. Clinical features of achondroplasia include short statue, prominent abdomen and buttocks, macrocephaly with frontal bossing and depressed nasal bridge, short upper and lower extremities and limited range of motion at the elbows. Hypochondroplasia, a milder form of achondroplasia, presents with short stature without macrocephaly and less pronounced short appendicular bones [149]. Type I and type II thanatophoric dysplasia are the two less common forms that present as extremely short limbs, redundant skin on the arms and legs and respiratory failure resulting in high mortality rate. Type I thanatophoric dysplasia is characterized by presence of flattened spines and curved femurs. Type II thanatophoric dysplasia is distinguished by straight femur and presence of characteristic skull abnormality named a cloverleaf skull [150,151,152]. 

The diagnosis of achondroplasia is mainly based on clinical examination and skeletal X-rays. Genetic testing is indicated in cases of uncertainty. Approximately 98% of patients with achondroplasia have a c.1138G > A mutation and around 1% have a c.1138G > C mutation, whereas 70–80% of patients with hypochondroplasia have c.1620C > A or c.1620C > G mutations [149,153]. 

#### 4.1.3. Treatment

There is no specific treatment that can reverse skeletal abnormalities due to *FGFR3* mutation. An attempt has been made to screening potential drugs to attenuate FGFR3 activation using a human disease-based system, but no agent has been identified to date [154]. 

### 4.2. Multiple Exostoses

#### 4.2.1. Overview and Pathogenesis

Multiple exostoses, also known as osteochondromatosis or diaphyseal acalsis, is an autosomal dominant genetic disorder caused by loss-of-function mutation of the *EXT1* and *EXT2* genes that encode Golgi-associated glycosyltransferases, which are responsible for heparan sulfate biosynthesis. These genes are also shown to be responsible for tumor suppression. The mutation results in an abnormal proliferation of growth plate cartilage [155,156].

#### 4.2.2. Clinical and Radiographic Presentation

Skeletal changes in multiple exostosis are usually asymptomatic and benign, with a minimal risk of malignant transformation into chondrosarcoma. Nevertheless, some osteochondroma lesions may interfere with joint functions or cause impingement of muscle tendons and nerves [157]. There are also reports on nontraumatic fractures of osteochondroma that either spontaneously healed or required surgical treatment [158,159,160]. The growth plate lesions stop growing when growth ceases but can sometimes resume during pregnancy. Radiographic features of multiple exostoses include single or multiple mass lesions located near the growth plate of the long bones. These lesions are in direct communication with the marrow cavity and are associated with local cortical bone resorption [157]. 

#### 4.2.3. Treatment

The mainstay treatment for symptomatic multiple exostoses is surgery. Extensive investigations have been performed to identify strategies to inhibit osteochondroma formation with potential therapeutic targets including modulation of BMP and hedgehog signaling pathways and inhibition of heparinase activity [156,161,162]. However, no specific treatment addressing defects in heparan sulfate synthesis has been approved for clinical use to date.

### 4.3. Patchydermoperiostosis

#### 4.3.1. Overview and Pathogenesis

Patchydermoperiostosis also called idiopathic hypertrophic osteoarthropathy is a rare genetic disorder commonly affecting young males characterized by digital clubbing, periostosis (abnormal proliferation of periosteal bone), pachydermia (skin thickening) and hyperhidrosis (excessive sweating) [163,164,165]. It is also known as Tourain-Solente-Gole syndrome, named after the three dermatologists who recognized this condition as a familial disorder [164,165,166]. The pathogenesis of patchydermoperiostosis is largely unknown but is thought to be associated with a mutation of the *HPGD* or *SLCO2A1* gene that encode 15-hydroxyprostaglandin dehydrogenase and solute carrier organic anion transporter family member 2A1, respectively. It is postulated that one of these genetic mutations can cause elevated prostaglandin E2 levels, which can subsequently cause tissue remodeling and vascular stimulation, leading to skin and skeletal abnormalities [167,168,169].

#### 4.3.2. Clinical Presentation

Patchydermoperiostosis usually manifests in a painless and self-limited course. Affected patients can present with only one or a few of the following features: clubbing of the fingers and toes, furrowing and thickening of the facial skin and the scalp, proliferating periosteum of the long bones causing cylindrical enlargement of upper and lower extremities and hyperhydrosis of the hands and feet. Joint involvement can sometimes occur and may cause significant discomfort [163,165]. 

#### 4.3.3. Treatment

Treatment for patchydermoperiostosis is primarily supportive and symptomatic. Joint pain can be managed with nonsteroidal anti-inflammatory drugs, colchicine or steroids [170]. In refractory cases, surgical vagotomy may be performed to decrease joint pain and swelling [171]. Retinoids can be used for treatment of skin involvements and plastic surgery may be considered to improve facial appearance [166,172]. 

### 4.4. Osteoporosis-Pseudoglioma Syndrome

#### 4.4.1. Overview and Pathogenesis

Osteoporosis-pseudoglioma syndrome (OPPG) is a rare autosomal recessive disorder characterized by severe osteoporosis and infancy-onset blindness [173]. The name pseudoglioma in this syndrome refers to a condition resembling retinal glioma on examination. OPPG is caused by loss-of-function mutations in the *LRP5* gene, which regulates bone metabolism by acting as a Wnt co-receptor. It also plays an essential low in development of the retinal vasculature [174,175,176]. 

#### 4.4.2. Clinical Presentation

Skeletal presentations of OPPG include juvenile-onset osteoporosis, fragility fractures, scoliosis, short stature, limb deformities and craniotabes [173]. Visual impairment in OPPG presents as premature arrest of retinal vasculature resulting in retinal avascularity, thereby causing retinal detachment and blindness in infanthood [177]. Other uncommon presentations of OPPG include mild intellectual disability, generalized hypotonia, joint hypermobility and seizures [178,179]. 

#### 4.4.3. Treatment

Little is known about the treatment strategies of skeletal involvement in OPPG. Bisphosphonates and denosumab have been shown to improve bone mineral density in case reports [179,180]. Teriparatide was also used in patients with OPPG who failed to respond to pamidronate [179,181]. 

### 4.5. Osteogenesis Imperfecta

#### 4.5.1. Overview and Pathogenesis

Osteogenesis imperfecta (OI), also known as ‘brittle bone disease’, is the most common hereditary metabolic bone disease that affects approximately 8 per 100,000 live births [182]. OI is a spectrum of genetic disorders associated with abnormalities in type I collagen, which is a major component of skin, bones, tendons, ligament and sclerae. Most of the patients with OI have autosomal dominant genetic variants in the *COL1A1* or *COL1A2* genes that encode the α1 and α2 chains of type 1 collagen, respectively [183]. Others have may have recessive forms of OI which are shown to be associated with genes involved in processing of type 1 collagen or osteoblast function [184]. It is also worth noting based on a previous report that up to about 10% of patients with clinical features of OI remained without genetic diagnosis [185]. 

#### 4.5.2. Clinical Presentation

OI can be classified into 22 categories with a wide variety of clinical spectrum ranging from mild phenotypes that manifest in adulthood to severe phenotypes that can cause in utero fetal demise [186,187]. The main skeletal features of OI include decreased bone mass, skeletal deformities, fragility fractures and growth defect. Other presentations may include blue or gray-blue sclerae, bleeding disorder due capillary fragility, aortic aneurysm, joint hypermobility, hearing loss, dentinogenesis imperfecta and central nervous system abnormalities such as Chiari malformation and hydrocephalus [188,189]. 

It is important to note that many presentations of OI can be overlapping with other connective tissue disorders such as Ehlers-Danlos syndrome and Marfan’s syndrome. These include skeletal and capillary fragility, large vessel insufficiency, blue sclera as well as joint hypermobility [13]. Therefore, clinical diagnosis of OI can sometimes be difficult to differentiate with other connective tissue disorders especially in those patients with OI features do not have confirmed genetic diagnosis [185].

#### 4.5.3. Treatment

All patients with OI should receive adequate vitamin D and calcium intake to optimize bone mineralization. In children with severe OI, bisphosphonates are shown to be safe and effective in increasing lumbar spine bone mineral density and reducing fracture risk [190,191]. Denosumab is also shown to improve lumbar spine bone mineral density without serious adverse consequences in a study of ten children with OI type VI, although more data are needed to determine long term benefit of the medication [192]. Other management considerations include physical therapy and surgery for fracture management, routine dental examination to prevent periodontal diseases and interval screening of vision and hearing [13].

### 4.6. Other Hereditary Connective Tissue Disorders including Ehlers-Danlos Syndrome, Marfan Syndrome and Loeys-Dietz Syndrome

#### 4.6.1. Overview and Pathogenesis

Besides OI, other hereditary connective tissue disorders, including Ehlers-Danlos syndrome (EDS), Marfan’s syndrome and Loeys-Dietz syndrome, are shown to be associated with skeletal fragility and therefore can be considered metabolic bone diseases [193,194,195,196,197]. EDS is a spectrum of disorders of collagen and elastin with 13 types with different causative genetic mutations, most of which were associated with joint hypermobility and skeletal and vascular fragility [198]. The most common type of EDS is hypermobility type (hEDS, or type 3 EDS), the only type with unknown causative genetic variant. 

Marfan’s syndrome is an autosomal dominant connective tissue disorder caused by a defect in elastic tissue homeostasis. Marfan’s syndrome type 1 is caused by mutations in the FBN1 gene that encodes the extracellular matrix protein fibrillin-1 protein. The mutated fibrillin-1 protein in lacks the ability to bind with transforming growth factor-β (TGF-β). Consequently, tissue TGF-β level increases and causes dysregulated homeostasis of microfibrils and elastic fibers, leading to multisystem manifestations including the syndromic Marfanoid body habitus with cardiovascular, musculoskeletal, skin and neurological abnormalities [199]. Marfan’s syndrome type 2 is caused by genetic mutation in the *TGFBR2* gene encoding the TGF-β receptor type 2 [200]. 

Loeys-Dietz syndrome (LDS) is another rare hereditary connective tissue disorder characterized by thoracic aortic aneurysm or dissection and widespread connective tissue involvement. LDS type 1–4 are caused by mutations of genes in the TGF-β signaling pathway (i.e., LDS type 1: TGFBR1; LDS type 2: TGFBR2; LDS type 3: SMAD3 encoding the TGF-β receptor cytoplasmic effector; LDS type 4: TGFB2 encoding the TGF-β2 ligand) [201]. LDS type 5 (Rienhoff syndrome) is caused by mutation in the TGFB3 gene that encodes a nonfunctional TGF-β3 ligand [202]. 

#### 4.6.2. Clinical Presentation and Diagnosis

Hereditary connective tissue disorders have a wide variety of skeletal, cardiovascular and skin presentation, as listed in Table 1. Given that the genetic basis of hEDS remains undetermined, diagnosis of hEDS is solely based on the Beighton’s clinical diagnostic criteria established in 1987, which include autosomal dominant inheritance pattern, skin involvement, generalized joint hypermobility, recurrent joint dislocation, chronic joint pain and positive family history [203]. In 2017, the International EDS Consortium updated the clinical diagnostic criteria for each type of EDS [198]. It is however worth noting that the new diagnostic criteria for hEDS criteria has become less sensitive and more specific than the Beighton’s 1987 criteria; without a diagnostic gold standard, this has made ascertainment of hEDS diagnosis become a topic of debate. Diagnosis of Marfan’s syndrome can be made based on the revised Ghent criteria followed by genetic testing. If a Marfan mutation is detected, testing in family members is recommended [204]. There is no specific clinical criteria for diagnosis of LDS and the diagnosis is generally confirmed by a molecular test [201].

With regard to skeletal health, hereditary connective tissue apart from OI are all shown to be associated with skeletal fragility due to uncertain pathogenesis. Many studies have demonstrated decreased BMD as well as increased risks of fracture in patients with EDS (including hEDS) [195,205,206], Marfan’s syndrome [193,194,207] and LDS [197]. Additionally, certain types of EDS are well-documented to cause severe skeletal deformities (i.e., type V, X, XIII, XV) [13]. It has also been reported in a case series of 72 infants with multiple fractures reported to be caused by non-accidental trauma that 93% (67 infants) had clinical evidence of EDS and/or at least one parent with hEDS based on the Beighton’s clinical diagnostic criteria [208]. Another report has demonstrated a pathogenic genetic mutation in the *CDCC134* gene which regulates collagen type 1 production in an infant with in utero multiple fractures with negative genetic test for OI whose mother had hEDS, supporting that the clinical presentations of hEDS and OI can overlap with genetic mutations not covered by clinically available genetic testing panel [209]. This condition has recently been recognized as OI type XXII [210]. 

#### 4.6.3. Treatment

The major component of management of hereditary connective tissue disorders is to identify patients at risk and perform active surveillance for cardiovascular abnormalities, which will not be discussed in this article. There is currently no specific treatment to reverse skeletal abnormalities in EDS, Marfan’s syndrome and LDS. Adequate calcium and vitamin D intake should be maintained to maximize bone mineralization and minimize fracture risk [13].

## 5. Conclusions

**Figure 2:**
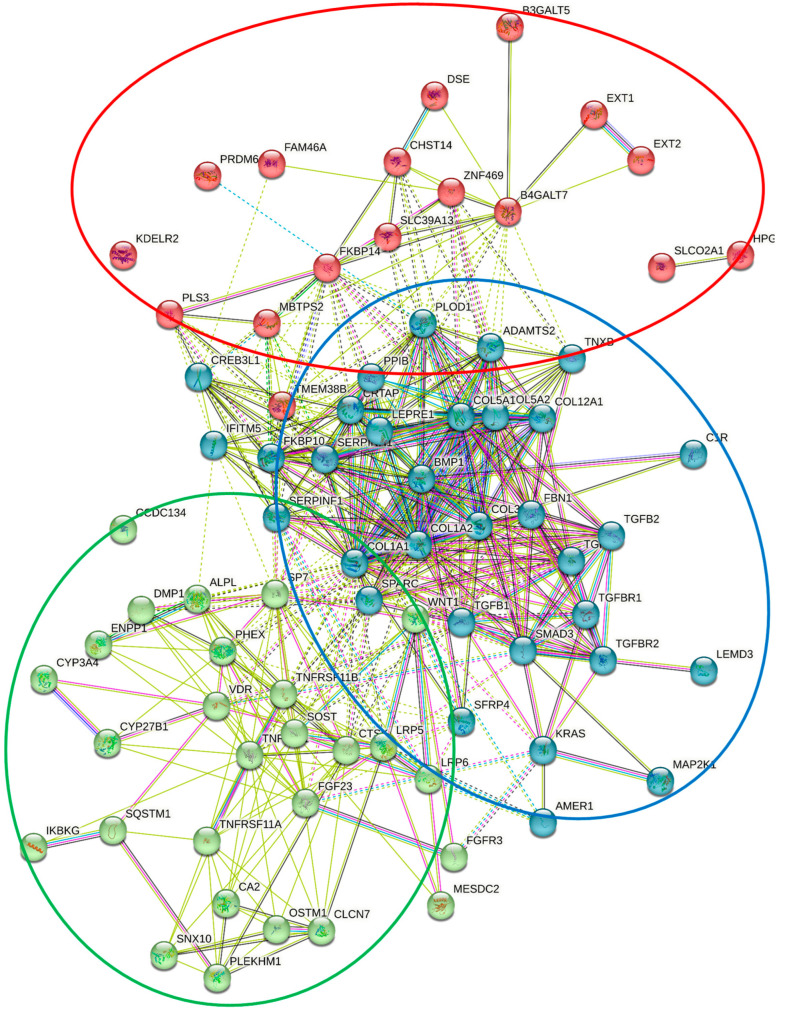
Protein–protein Interaction (PPI) Network was constructed from 78 genes contributing to hereditary metabolic bone diseases.

In this review, we examine a number of selected hereditary metabolic bone diseases and recognized causative genetic variations of 78 genes are responsible for these hereditary metabolic bone diseases. The clinical manifestations and causative genetic variations of these 78 genes that contributing to hereditary metabolic bone diseases were shown in Table 1. The proteins of these 78 genes were mapped to the STRING database and screened for significant interactions and PPI enrichment *p*-value was < 1.0 × 10^−16^ (Figure 2). This network has expected more interaction than random chance. This means that these proteins have more interactions among themselves than what would be expected for a random set of proteins of the same size and degree distribution drawn from the genome. Such an enrichment indicates that the proteins are at least partially biologically connected, as a group.

These biological connections compose 3 clusters. These clusters are involved in 3 major pathways includes (1). biosynthesis, processing and secretion of extracellular matrix, (2). metabolic functions and activity of PTH, vitamin D and FGF23 and (3). osification and, bone mineralization processes. Mutations of the genes in this cluster can have a direct or indirect influence not only on their tissue expression and expression of other proteins but also can dramatically effect osteoclast, osteoblast and osteochondroma cell proliferation and differentiation. 

Among the 78 genes discussed in our review, 17 genes were found to be associated with osteoporosis based on the data from genome-wide association study by Morris et al. [211]. These genes include: *ALPL*, *COL1A1*, *COL1A2*, *DMP1*, *LRP5*, *LRP6*, *SFRP4*, *SMAD3*, *SOST*, *SP7*, *TCIRG1*, *TGFB2*, *TGFBR2*, *TNFRSF11A*, *TNFRSF11B*, *TNFSF11* and *WNT1*.

The 78 genes contributing to hereditary metabolic bone diseases were shown in Table 1. The proteins of these genes were mapped to the STRING database and screened for significant interactions and PPI enrichment *p*-value was < 1.0 × 10^−16^. 

This network has significantly more interactions than expected. This means that these proteins have more interactions among themselves than what would be expected for a random set of proteins of the same size and degree distribution drawn from the genome. Such an enrichment indicates that the proteins are at least partially biologically connected, as a group.

These biological connections compose 3 clusters. These clusters have been shown by the red, blue and green circles. The blue cluster is the largest cluster includes 33 proteins that they are involved in collagen and fibrillar collagen trimer, collagen biosynthetic process, extracellular matrix constituent secretion and Glycosaminoglycan biosynthesis. Mutations of the gens in this cluster can influence on their tissue expression specifically in osteochondroma cells. The red cluster related to different types of Osteogenesis imperfecta, Ehlers-Danlos syndrome and Loeys-Dietz syndrome. 

The green cluster includes 28 proteins that they are involved in parathyroid hormone synthesis, NF-kappa B signaling pathway, secretion and action, Response to vitamin D, Vitamin d metabolic process, Ossification, Bone mineralization, Vitamin D receptor and FGF23 signaling pathway. Mutations of the gens in this cluster can influence on their tissue expression specifically in Osteoclast proliferation and differentiation. The green cluster related to different types of Rickets, Hypophosphatemia, Osteosclerosis, Osteopetrosis, Osteoporosis, Paget’s disease and Bone remodeling disease. 

The red cluster includes 17 proteins that they are involved in Glycosaminoglycan biosynthetic process. Mutations of the gens in this cluster can influence on their tissue expression specifically in Osteochondroma cell. The blue cluster related to different types of Hereditary multiple exostoses, Osteochondrodysplasia, Ehlers-Danlos syndrome and Osteogenesis imperfecta. Copyright Holick 2022.

## Figures and Tables

**Table 1:** Clinical manifestations and causative genetic variations of hereditary metabolic bone diseases.

Disorder	Clinical Manifestations	Causative Genetic Variations
Sclerosing disorders		
Autosomal dominant osteopetrosis	Increased BMD, bony sclerosis, bone fragility, metaphyseal deformity, osteomyelitis, tooth eruption defects, dental caries, moderate bone marrow failure, cranial nerves impingement (II, VII, VIII)	- Type I: *LRP5* mutation - Type II: *CLCN7* mutation
Classic autosomal recessive osteopetrosis	Increased BMD, bony sclerosis, bone fragility, metaphyseal deformity, osteomyelitis, tooth eruption defects, dental caries, hydrocephalus, hypocalcemia, severe bone marrow failure, extramedullary hematopoiesis, hepatosplenomegaly, cranial nerves impingement (II, VII, VIII)	- Osteoclast-rich autosomal recessive osteopetrosis: *TCIRG1*, *CLCN7*, *OSTM1*, *SNX10*, or *PLEKHM1* mutation - Osteoclast-poor autosomal recessive osteopetrosis: *TNFSF11* (encoding RANKL) or *TNFRSF11A* (encoding RANK) mutation
X-linked osteopetrosis, lymphedema, anhidrotic ectodermal dysplasia and immunodeficiency	Increased BMD, bony sclerosis, bone fragility, metaphyseal deformity, osteomyelitis, tooth eruption defects, dental caries, anhidrotic ectodermal dysplasia, lymphedema, immunodeficiency	- *IKBKG* stop codon mutation
Autosomal recessive osteopetrosis with renal tubular acidosis	Increased BMD, bony sclerosis, bone fragility, metaphyseal deformity, osteomyelitis, tooth eruption defects, dental caries, renal tubular acidosis, developmental delay, intracranial calcification, cranial nerves impingement, bone marrow failure (rare)	- *CAII* mutation
Progressive Diaphyseal Dysplasia	Symmetric periosteal/endosteal thickening of long bone diaphysis (primarily femur and tibia), increased BMD, leg pain, muscle weakness, fatigue, slim limbs, tender bones, cranial enlargement, prominent forehead, cranial nerve palsy, hydrocephalus, hypocalciuria, hypocalcemia	- *TGFβ1* mutation
Melorheostosis	Bone resembles dripping wax from a melting candle (affecting appendicular skeleton and adjacent soft tissue), dense hyperostosis of periosteal/endosteal surfaces, pain, bony swelling, joint and limb deformities, limited motion, numbness, weakness	- *MAP2K1* mutation - Mutations in KRAS pathways (minority of cases)
Juvenile Paget’s Disease	Rapid bone turnover in children, bone deformities and fractures, short stature, elevated bone alkaline phosphatase, hearing loss, retinopathy, vascular calcification, internal carotid artery aneurysm	- *TNFRSF11B* mutation (encoding OPG)
Paget’s disease	Bone pain, fractures and deformity, headache, hearing loss, nerve compression, spinal stenosis, high-output cardiac failure	- *SQSTM1* mutation - *TNFRSF11A* (encoding RANK) - *TNFRSF11B* mutation (encoding OPG)
High bone mass associated with *LRP5* mutation	Extremely high BMD, increased calvarial thickness, craniosynostosis, striking square jaw, torus palatinus, thickened cortices of long bone	- *LRP5* mutation
High bone mass associated with *LRP6* mutation	Extremely high BMD, absence of adult maxillary incisors, broad jaw, torus palatinus, thickening of the skull, optic nerve dilatation, narrowing of optic and autidory canals	- *LRP6* mutation
Pyle disease	Genu valgum, “Erlenmeyer flask” deformity, metaphyseal fracture, dental abnormalities, prognathism	- *SFRP4* mutation
Hyperostosis Corticalis Generalista	Endosteal hyperostosis of the mandible, skull, ribs, clavicles, and diaphysis of long bones, facial nerve palsy, hearing loss, optic atrophy	- *SOST* mutation (encoding sclerostin)
Sclerosteosis	Generalized bone overgrowth, jaw enlargement, facial abnormality, cranial nerve impingement, increased intracranial pressure, syndactyly, tall stature	- *SOST* mutation
Pyknodysostosis	short-limbed, short stature, dysmorphic facial features (small jaw, obtuse mandibular angle, and convex nasal ridge), osteosclerosis, bone fragility and fractures, dental and nail abnormalities, kyphoscoliosis, chest deformity, high arched palate, proptosis, blue sclera	- *CTSK* mutation (encoding cathepsin K)
Osteopathia Striata	Linear striations within the metaphyseal areas of long bones, macrocephaly, characteristic facial features (frontal bossing, hypertelorism, depressed nasal bridge, prominent mandible, and epicanthal folds), hearing loss, orofacial clefting, mild developmental delay In males: congenital, musculoskeletal defects (in mild cases). Multiple-malformation syndrome (in severe cases)	- *WTX* mutation
Osteopoikilosis	Numerous bone islands that typically affect the appendicular skeleton, usually free of any major symptoms	- *LEMD3* mutation
Demineralization disorders		
Hypophosphatasia		
Odonto hypophosphatasia	Premature loss of primary teeth	- *ALPL* mutation
Adult hypophosphatasia	Bone pain, pseudogout, calcium pyrophosphate dihydrate crystal deposition in ligaments, and soft tissues.	
Childhood hypophosphatasia	Premature loss of primary teeth, bone pain, craniosynostosis, rachitic rosary, flaring of metaphysis, bowed legs, waddling gait	
Infantile hypophosphatasia	Skeletal rachitic deformities, brachycephaly, hypertelorism, increased intracranial pressure, tracheomalacia, chest wall deformity, recurrent pneumonia, hypercalcemia, hypercalciuria, pyridoxine-dependent seizure	
Perinatal hypophosphatasia	In utero limb deformities, cardiopulmonary failure, brain hemorrhage, myelophthisic anemia	
Hypophosphatemic rickets	Proximal muscle weakness, waddling gait, short stature, defective limb growth with preserved trunk growth, delayed tooth eruption, increased risk of dental abscesses, frontal bossing, parietal flattening, craniosynostosis, genu valgum/varum, intoeing or extoeing leg deformities, thickening of costochondral junctions	- X-linked hypophosphatemic rickets: *PHEX* mutation - Autosomal dominant hypophosphatemic rickets: *FGF23* mutation - Autosomal recessive hypophosphatemic rickets type 1: *DMP1* mutation - Autosomal recessive hypophosphatemic rickets type 2: *ENPP1* mutation - Autosomal recessive hypophosphatemic rickets type 1: *DMP1* mutation
Vitamin D-dependent rickets	Proximal muscle weakness, waddling gait, short stature, sweating, delayed tooth eruption, sweating, craniotabes, frontal bossing, widened fontanelles, rachitic rosary, sternal protrusion, ribs deformities, flattened pelvic bones, bowing deformities of arms and legs, genu valgum/varum, flattened pelvic bones, hypocalcemic tetany, seizures, laryngospasm, cardiomyopathy, alopecia (for vitamin D-dependent rickets type II)	- Vitamin D dependent rickets type I: *CYP27B1* mutation - Vitamin D dependent rickets type II: VDR mutation - Vitamin D dependent rickets type III: *CYP3A4* mutation - Reported case: abnormal expression of hormone responsive element-binding protein that binds to the vitamin D responsive element
Axial osteomalacia	Bone pain along the axial skeleton, findings resembling ankylosing spondylosis	- Unknown genetic mutation
Disorders of bone matrix and cartilage formation		
Achondroplasia	Short statue, prominent abdomen and buttocks, macrocephaly, frontal bossing, depressed nasal bridge, short extremities, limited range of motion at the elbows	- *FGFR3* mutation
Hypochondroplasia	Short stature without macrocephaly, short appendicular bone (less pronounced than achondroplasia)	
Thanatophoric dysplasia	Short extremities, redundant skin on the arms and legs, respiratory failure, flattened spine and curved femurs (type 1), straight femur and cloverleaf skull (type 2)	
Multiple exostosis	Multiple osteochondroma causing impingement of nerves and muscle tendons, nontraumatic fractures of osteochondroma	- *EXT1* or *EXT2* mutation
Patchydermoperiostosis	Clubbing of the fingers and toes, furrowing and thickening of the facial skin and the scalp, cylindrical enlargement of upper and lower extremities, hyperhydrosis, arthalgia	- *HPGD* or *SLCO2A1* mutation
Osteoporosis-pseudoglioma syndrome	Juvenile-onset osteoporosis, fragility fractures, scoliosis, short stature, limb deformities, craniotabes, visual impairment, intellectual disability, hypotonia, joint hypermobility, seizures	- *LRP5* mutation
Osteogenesis imperfecta		
OI type I	Mild, normal or short stature, blue sclerae, late-onset hearing loss	- Autosomal dominant: *COL1A1* or *COL1A2* mutation - X-linked: *PLS3* mutation
OI type II	Perinatally lethal, minimal calvarial mineralization	- *COL1A1*, *COL1A2*, *CRTAP*, *LEPRE1*, *PPIB* or *BMP1* mutation
OI type III	Severe, progressively deforming bones	- *COL1A1*, *COL1A2*, *CRTAP*, *LEPRE1*, *PPIB*, *FKBP10*, *SERPINH1*, *SERINF1* or WNT1 mutation
OI type IV	More severe than type I, lesser severe than type II and type III, short stature, bone deformity	- *COL1A1*, *COL1A2*, *CRTAP*, *FKBP10*, *SP7*, *SERPINF1*, *WNT1* or *TMEM38B* mutation
OI type V	Normal-to-severe skeletal deformity, hyperplastic callus formation, intraosseous membrane ossifications	- *IFITM5* mutation
OI type VI	Presence of osteoid, fish-scale appearance of the lamellar bone pattern	- *SERPINF1* mutation
OI type VII	Severe to lethal, rhizomelia	- *CRTAP* mutation
OI type VIII	Severe to lethal, rhizomelia, coxa vara, popcorn metaphyses	- *P3H1* mutation
OI type IX	Short bowed femurs with anterior, bowing of the tibiae, grey sclerae	- *PPIB* mutation
OI type X	Severe skeletal deformity, blue sclerae, dentinogenesis imperfecta, skin abnormalities, inguinal hernia	- *SERPINH1* mutation
OI type XI	Joint contractures (distorted lamellar, structure and a fish scale-like pattern), normal to grey sclerae	- *FKBP10* mutation
OI type XII	Fractures, mild bone deformations, generalized osteoporosis, delayed teeth eruption, progressive hearing loss, no dentinogenesis imperfecta, white sclerae	- *SP7* mutation
OI type XIII	Severe skeletal deformity, delayed tooth eruption, facial hypoplasia	- *BMP1* mutation
OI type XIV	Severe bone deformity, normal-to-blue sclerae	- *TMEM38B* mutation
OI type XV	Fractures, bone deformities, short stature, blue sclerae	- *WNT1* mutation
OI type XVI	Prenatal onset of multiple fractures of ribs and long bones, blue sclerae, decreased ossification of the skull, and severe demineralization.	- *CREB3L1* mutation
OI type XVII	Fractures, motor delay, muscle hypotonia, lower extremity weakness, decreased calf muscle mass, joint hyperlaxity, and soft skin	- *SPARC* mutation
OI type XVIII	Congenital bowing of the long bones, wormian bones, blue sclerae, vertebral collapse, multiple fractures	- *TENT5A* mutation
OI type XIX	Moderate short stature, blue sclerae, pectus carinatum, bowing of lower extremity long bones, multiple fractures	- *MBTPS2* mutation
OI type XX	Osteopenia, skeletal deformity, multiple fractures, respiratory failure	- *MESD* mutation
OI type XXI	Short stature, failure to thrive, wormian bones, bowed limbs, chest deformity, hypotonia, joint hypermobility, dysmorphic facies, blue sclerae, dentinogenesis imperfecta, scoliosis, fractures, platyspondyly.	- *KDELR2* mutation
OI type XXII	Intrauterine growth retardation, short stature, multiple fractures, decreased thoracic size, short limbs, blue sclerae	- *CCDC134* mutation
Ehlers-Danlos syndrome		
Classical EDS, types I and II	Joint hypermobility, hyperextensible skin, easy bruisability, doughy-velvety skin, atrophic scars	- *COL5A1*, *COL5A2* or *COL1A1* (rarely) mutation
Classical-like EDS	Hyperextensible skin, doughy-velvety skin texture, atropic scars, easy bruisability, joint hypermobility	- *TNXB* mutation
Hypermobile EDS, type III	Joint hypermobility, joint dislocations-subluxations including temporal mandibular joint, hyperextensible skin, doughy-velvety skin, hernias, gastroparesis, high palate with dental crowding, bone fractures, vascular fragility, mast cell hyperactivity, postural orthostatic tachycardia syndrome, atrophic and hypertrophic scarring, poor wound healing, pieziogenic blisters on heels	- Unknown genetic mutation
Vascular EDS, type IV	Arterial rupture (aorta, mesenteric, cerebrovascular, splenic, renal arteries), organ rupture (colon, uterus), easy bruising, translucent skin	- *COL3A1* or *COL1A1* (rarely) mutation
Cardiac-valvular type EDS	Progressive weakening of heart valves, hyperextensible skin, atrophic scars, easy bruisability, joint hypermobility	- *COL1A2* mutation
Kyphoscoliotic EDS, type VI	Joint hypermobility, kyphoscoliosis, osteopenia, hypotonia at birth, blue sclerae, Marfanoid habitus	- *PLOD1* or *FKBP14* mutation
Arthrochalasia EDS, types VIIA and VIIB	Congenital hip dislocations, recurrent subluxations, joint hypermobility, hyperextensible skin, muscle hypotonia, osteopenia	- *COL1A1* or *COL1A2* mutation
Dermatosparaxis EDS, type VIIC	Severe bruisability, blue sclerae, severe skin fragility, sagging skin, visceral fragility, growth retardation	- *ADAMTS2* mutation
Periodontal EDS, type VIII	Early onset severe periodontitis, unattached gingiva, pretibial plaques, hyperextensible skin, Marfanoid features, joint hypermobility	- *C1R* mutation
Spondylodysplastic EDS	Short stature, delayed eruption of teeth, hypodontia, limb bowing, joint laxity, osteopenia, hyperextensible, thin skin, delayed wound healing with atrophic scars	- *B4GALT7*, *B3GALT5* or *SLC39A13* mutation
Musculocontractural EDS	Congenital contractures (thumb, finger, club feet), severe kyphoscoliosis, recurrent dislocations, easy bruisability, craniofacial features (broad forehead, small mouth, micrognathia, protruding jaw), hyperextensible fragile skin, atrophic scars	- *CHST14* or *DSE* mutation
Brittle cornea syndrome	Thin cornea, retinal detachment, globe rupture, blue sclerae, keratoconus, high myopia	- *ZNF469* or *PRDM6* mutation
Myopathic EDS	Congenital hypotonia, proximal joint contractures, distal joint hypermobility, atrophic scars	- *COL12A1* mutation
Marfan’s syndrome	Aortic disease: aortic root disease, leading to aneurysmal dilatation, aortic regurgitation and dissection; Cardiac disease: mitral valve prolapse; Skeletal findings: arachnodactyly, pectus carinatum, pectus excavatum, abnormal upper/lower segments and arm span/height, scoliosis, kyphosis, malar hypoplasia, retrognathia; Ocular abnormalities: ectopia lentis, flat cornea, miosis, retinal detachment, glaucoma. Dural ectasia, emphysematous, pneumothorax, skin striae, arm span to height ratio >1.5	- Type 1: *FBN1* mutation - Type 2: *TGFBR2* mutation
Loeys-Dietz syndrome	Craniosynostosis, scoliosis, pectus excavatum/carinatum, clubfoot, pes planus, elongated limbs, joint instability/contracture, dural ectasia, bruising, abnormal scar, striae, skin translucency, spontaneous pneumothorax, hernias, hypertelorism, strabismus, bifid uvula, cleft palate, increased risk of immune disorders (i.e., allergies, asthma, eczema, inflammatory bowel disease)	- Type 1: *TGFBR1* mutation - Type 2: *TGFBR2* mutation - Type 3: *SMAD3* mutation - Type 4: *TGFB2* mutation - Type 5: *TGFB3* mutation

Abbreviations: BMD: Bone mineral density; EDS: Ehlers-Danlos syndrome; OI: Osteogenesis imperfecta; OPG: osteoprotegerin; RANKL: Receptor activator of nuclear factor kappa-Β ligand; RANK: Receptor activator of nuclear factor kappa-Β. Copyright Holick 2022.

## Data Availability

Not applicable.
